# QTL mapping for field resistance to wheat blast in the Caninde#1/Alondra population

**DOI:** 10.1007/s00122-020-03624-x

**Published:** 2020-06-02

**Authors:** Xinyao He, Muhammad Rezaul Kabir, Krishna K. Roy, Md. Babul Anwar, Kaijie Xu, Felix Marza, Firuz Odilbekov, Aakash Chawade, Etienne Duveiller, Eric Huttner, Pawan K. Singh

**Affiliations:** 1 International Maize and Wheat Improvement Center (CIMMYT), Apdo. Postal 6-641, 06600 Mexico, DF Mexico; 2 Bangladesh Wheat and Maize Research Institute (BWMRI), Nashipur, Dinajpur, Bangladesh; 3 Institute of Cotton Research, Chinese Academy of Agricultural Sciences, Anyang, Henan China; 4 Instituto Nacional de Innovación Agropecuaria y Forestal (INIAF), La Paz, Bolivia; 5 Department of Plant Breeding, Swedish University of Agricultural Sciences, 23053 Alnarp, Sweden; 6 Australian Centre for International Agricultural Research, 38 Thynne St, Bruce, ACT 2617 Australia

## Abstract

**Key message:**

Wheat blast resistance in Caninde#1 is controlled by a major QTL on 2NS/2AS translocation and multiple minor QTL in an additive mode.

**Abstract:**

Wheat blast (WB) is a devastating disease in South America, and it recently also emerged in Bangladesh. Host resistance to WB has relied heavily on the 2NS/2AS translocation, but the responsible QTL has not been mapped and its phenotypic effects in different environments have not been reported. In the current study, a recombinant inbred line population with 298 progenies was generated, with the female and male parents being Caninde#1 (with 2NS) and Alondra (without 2NS), respectively. Phenotyping was carried out in two locations in Bolivia, namely Quirusillas and Okinawa, and one location in Bangladesh, Jashore, with two sowing dates in each of the two cropping seasons in each location, during the years 2017–2019. Genotyping was performed with the DArTseq® technology along with five previously reported STS markers in the 2NS region. QTL mapping identified a major and consistent QTL on 2NS/2AS region, explaining between 22.4 and 50.1% of the phenotypic variation in different environments. Additional QTL were detected on chromosomes 1AS, 2BL, 3AL, 4BS, 4DL and 7BS, all additive to the 2NS QTL and showing phenotypic effects less than 10%. Two codominant STS markers, *WGGB156* and *WGGB159*, were linked proximally to the 2NS/2AS QTL with a genetic distance of 0.9 cM, being potentially useful in marker-assisted selection.

**Electronic supplementary material:**

The online version of this article (10.1007/s00122-020-03624-x) contains supplementary material, which is available to authorized users.

## Introduction

Wheat blast (WB) is an emerging and devastating disease of wheat, caused by the fungus *Magnaporthe oryzae* pathotype *Triticum* (MoT), leading to yield losses of up to 100% and grain quality deterioration, greatly threatening food security in the epidemic regions (Kohli et al. 2011; Cruz and Valent 2017). This disease first appeared in 1985 in the Paraná state of Brazil and soon spread throughout many of the important wheat-producing areas in Brazil (lgarashi et al. 1986; Goulart and Paiva 1990; Picinini and Fernandes 1990; Dos Anjos et al. 1996). Afterward, WB gradually spread to the neighboring countries of Bolivia, Paraguay and Argentina (Barea and Toledo 1996; Viedma 2005; Perelló et al. 2015) and had been confined to South America until recently. In 2016, WB was found in Bangladesh, which was its first outbreak outside South America (Malaker et al. 2016), causing yield reduction of up to 51% in some epidemic regions (Islam et al. 2016). This confirmed the risk identified by Duveiller et al. (2011) based on the similarity of agro-climatic conditions in Bangladesh and parts of South America, following the severe epidemics in South America in 2009. Therefore, WB became a serious threat to the neighboring countries of Bangladesh. WB-vulnerable areas amounting to 7 million ha in India, Pakistan and Bangladesh were identified, with an estimated potential annual yield loss of about 0.89–1.77 million tons (Mottaleb et al. 2018). With global warming, this disease might further spread to other major wheat production regions like USA, Ethiopia or Australia (Cao et al. 2011; Maciel 2011; Cruz et al. 2016a).

The pathogen has a global distribution and is regarded as the most devastating fungal pathogen worldwide (Dean et al. 2012). *M. oryzae* is a hemibiotrophic ascomycetous fungal species, having a series of pathotypes, among which are the widely distributed rice pathotype MoO and the wheat pathotype MoT (Maciel 2011; Cruz and Valent 2017). Unlike rice blast, for which research has been ongoing for many decades, WB is a relatively new disease and there are large knowledge gaps in terms of epidemiology, host–pathogen interaction, management, etc. (Duveiller et al. 2016; Cruz and Valent 2017). So far, control of WB relies essentially on fungicide applications. However, the low effectiveness of fungicide application under high disease pressure (Fernandes et al. 2017) and the high risk of development of fungal resistance to fungicides (Castroagudín et al. 2015) are two major limitations of this approach. Host resistance is another major WB management component and is more economical and environmentally friendly compared to fungicide application. Since the first WB outbreak in Brazil in the 1980s, searches for sources of resistance have been carried out, but many the resistant lines identified in the early days became susceptible, due to the fast evolution of MoT (Duveiller et al. 2016). Nevertheless, varieties showing lasting resistance or tolerance to WB in South America have been identified: BR 18 and CD 116 in Brazil, Caninde#1 and Itapua 75 in Paraguay, and Montacu and Urubo in Bolivia (Ha et al. 2012; Buerstmayr et al. 2017). Many of those lines were derived from the CIMMYT wheat line Milan and have the 2NS/2AS translocation that is further discussed.

So far, most genetic studies for WB resistance were performed at the seedling stage, where the host–pathogen interaction appeared to follow the gene-for-gene model (Anh et al. 2015). The resistance genes identified in such experiments are *Rmg2*, *Rmg3*, *Rmg7*, *Rmg8*, *RmgTd(t)* and *RmgGR119* (Zhan et al. 2008; Cumagun et al. 2014; Tagle et al. 2015; Cruz et al. 2016b; Wang et al. 2018a) for host resistance against MoT, and *Rmg1*, *Rmg4*, *Rmg5*, *Rmg6* (Hau et al. 2007; Nga et al. 2009; Vy et al. 2014) for resistance against non-MoT strains of *M. oryzae*. Of these genes, *Rmg7*, *Rmg8* and *RmgGR119* also conferred resistance at the adult plant stage against spike infection by MoT, but Cruz and Valent (2017) pointed out that the resistance conferred by *Rmg7* has been overcome by recent MoT isolates, whereas *Rmg8* and *RmgGR119* remain to be tested with the new MoT isolates.

Genetics for field WB resistance is much less researched compared to that for seedling resistance, but it is generally accepted that quantitative resistance predominates in this pathosystem, as a continuous variation was observed among the evaluated germplasm (Maciel et al. 2014; Cruz et al. 2016b). So far, the only known effective field WB resistance source is 2NS/2AS translocation. The 2NS chromosomal segment was introduced from *Aegilops ventricosa* (Zhuk.) to replace the distal region of 2AS in wheat, in order to utilize the rust resistance genes *Lr37*, *Sr38*, and *Yr17* (Helguera et al. 2003). Later, it was found that other resistance genes, including *Cre5* for cereal cyst nematode resistance (Jahier et al. 2001) and *Rkn3* for root-knot nematodes resistance (Williamson et al. 2013), are present in this segment. Recently, Cruz et al. (2016b) reported the significant effects of 2NS in WB resistance, which conferred 64–81% reduction in head blast severity in both spring and winter wheat. Juliana et al. (2019) further demonstrated the important role of 2NS in conferring WB resistance in CIMMYT germplasm, along with an additional finding that the 2NS lines have a significant yield advantage.

The main objectives of the current study were to map QTL for field WB resistance in a bread wheat recombinant inbred line (RIL) population Caninde#1/Alondra and to identify molecular markers linked to the QTL for their potential use in marker-assisted selection (MAS).

## Materials and methods

### Plant material

A RIL population of 298 F_2:7_ progenies, derived from a cross between Caninde#1 and Alondra, was developed by single seed descend. The female parent Caninde#1 has a pedigree of Milan/Munia, carries the 2NS/2AS translocation and shows consistently good WB resistance (Kohli et al. 2011). The male parent Alondra has a pedigree of D-6301/NAINARI-60//WEIQUE/RED-MACE/3/CIANO-F-67*2/CHRIS, does not carry the 2NS/2AS translocation and is susceptible to WB.

### Inoculum preparation

The protocol was modified from Cruz et al. (2016b). A fungal plug of 5 mm was taken from a Petri dish culturing the MoT isolate and was then transferred onto plates with oatmeal agar that was prepared following the below steps. Fifty grams of rolled oats was added into 800 ml of distilled water, which was boiled for 5 min with a magnetic stirrer, filtered through four layers of cheesecloth and adjusted to 1 L with distilled water. Finally, 15 g of agar was added before autoclave. Agar plates with the MoT isolate were incubated under 18–28 °C with 12 h of light/dark photoperiod. Seven days later, mycelium grown on the plates was scraped off with a spatula and the plates were cultivated for three more days under continuous light to induce sporulation. Upon conidia harvest, the plates were flooded with 10 ml of autoclaved distilled water and gently scraped with a brush. The spore suspension was collected in a test tube and was vortexed briefly to separate conidia and mycelia, and the latter was discarded via filtering through two layers of cheesecloth. The inoculum was adjusted to 80,000 conidia/ml with a hemocytometer under a microscope, and then, Tween-20 was added to make a concentration of 0.02% for field application.

### Field experiments

Field trials took place in three locations, i.e., Quirusillas and Okinawa in Bolivia and Jashore in Bangladesh. Quirusillas is in the high land region of the Department of Santa Cruz, Bolivia, at an altitude of 1496 m above sea level (masl), with a cropping cycle from December to April. Okinawa is located in the lowland region of the Department of Santa Cruz, Bolivia, at an altitude of 267 masl, where the cropping cycle is from May to August. Jashore is in the southwestern region of Bangladesh, at an altitude of 7 masl, with a cropping season from December to April. The population was evaluated in two cropping cycles in each of the three locations, i.e., the 2017–2018 and 2018–2019 cycles in Quirusillas and Jashore and the 2018 and 2019 cycles in Okinawa, with two sowing dates (10 days difference) in each cropping cycle to expose the population to different environments. The experiments were then named according to the location, cropping cycle and sowing date, where ‘Quir’ stands for Quirusillas, ‘Jash’ for Jashore and ‘Oki’ for Okinawa, ‘18′ and ‘19′ for 2017–2018 or 2018 cycle and 2018–2019 or 2019 cycle, respectively, and ‘a’ and ‘b’ for the first and second sowing, respectively. For example, the experiment ‘Quir19b’ represents the second sown experiment carried out in Quirusillas in the 2018–2019 cycle.

The materials were sown in 1-m double rows spaced 20 cm apart, and no replication was made within each sowing. In Bolivia, Urubo and Atlax were used as resistant and susceptible checks, respectively, whereas in Bangladesh, the corresponding checks were BARI Gom 33 and BARI Gom 26. A misting system was set up in the nurseries, working from 8am to 7 pm, with 10 min of spraying each hour to keep a humid microenvironment that is conducive for WB infection. A mixture of locally collected MoT isolates was used for field inoculation, including OKI1503, OKI1704, QUI1505, QUI1601, QUI1612 in Bolivia and BHO17001, MEH17003, GOP17001.2, RAJ17001, CHU16001.3, JES16001 in Bangladesh. The isolates were selected based on their capacity of high sporulation. Inoculation was targeted to the anthesis stage of each line and was repeated 2 days later, where the inoculum was applied in the evening at a concentration of 80,000 spores/mL, using a CO_2_-driven backpack sprayer. WB evaluation was performed at 14 or 21 days after the first inoculation, depending on the disease development, on 10 spikes that had been tagged at anthesis. Upon evaluation, the total and infected number of spikelets were counted for each of the 10 spikes, and then WB index was calculated with the formula WB index = *Incidence* × *Severity*, where *Incidence* stands for the percentage of spike with WB symptom and *Severity* for the averaged percentage of infected spikelets. Days to heading (DH) and plant height (PH) were scored in all the experiments.

### Statistical analysis

The SAS program ver. 9.2 was used to conduct analysis of variance (ANOVA), with its PROC GLM module, whereas the calculation of Pearson's correlation coefficients was carried out using the PROC CORR function. The ANOVA results were used for calculating the heritability estimates, with the formula *H*^*2*^ = ${\sigma }_{\rm g}^{2}$/(${\sigma }_{\rm g}^{2}$+${\sigma }_{\rm g*y}^{2}$/*y* + ${\sigma }_{\rm g*s}^{2}$/*s* + ${\sigma }_{\rm e}^{2}$/*sy*), where ${\sigma }_{\rm g}^{2}$ represents genetic variance, ${\sigma }_{\rm g*y}^{2}$ for genotype-by-year interaction,${\sigma }_{\rm g*s}^{2}$ for genotype-by-sowing interaction, ${\sigma }_{\rm e}^{2}$ for error variance, *y* for the number of years and *s* for the number of sowing dates.

### Genotyping

Genomic DNA was extracted from young leaves of 2-week-old plants with the CTAB method. RILs of the population were genotyped with the DArTseq^®^ technology at the Genetic Analysis Service for Agriculture (SAGA) at CIMMYT, Mexico. Additionally, five STS markers in the 2NS/2AS region were used in this study, and they are *Ventriup-LN2* developed by Helguera et al. (2003), *WGGB156* and *WGGB159* by Wang et al. (2018b), *IWB11136* by Xue et al. (2018) and *cslVrgal3* that was derived from a follow-up study of Seah et al. (2001) (E. Lagudah, pers. comm.). Markers with more than 20% missing data points were removed from further analysis, as well as those highly distorted with a minor allele frequency less than 30%. Redundant markers identified with the BIN module of the ICIMapping v. 4.1 software (www.isbreeding.net) were discarded.

### Linkage and QTL mapping

Linkage groups (LG) were generated with the JoinMap v.4 software (Van Ooijen 2006), with LOD scores from 5 to 10 for grouping and the maximum likelihood algorithm for ordering within each LG. Chromosome anchoring of LGs was obtained via BLASTing sequences of the DArTseq markers against the Chinese Spring genome (IWGSC RefSeq v1.0). QTL analysis was conducted with MapQTL v6.0 (Van Ooijen 2009), in which interval mapping (IM) was first tried to detect potential QTL for a trait as well as the most closely linked markers to those QTL. Subsequently, multiple QTL mapping (MQM) for each QTL was carried out, using the tightly linked markers as cofactors. Significant QTL were defined in this study when they have a LOD score of > 3.0 in at least one environment or over the threshold of 2.0 in multiple environments. The software MapChart ver. 2.3 (Voorrips 2002) was used to draw LGs and LOD curves.

## Results

### Phenotyping

**Fig. 1:**
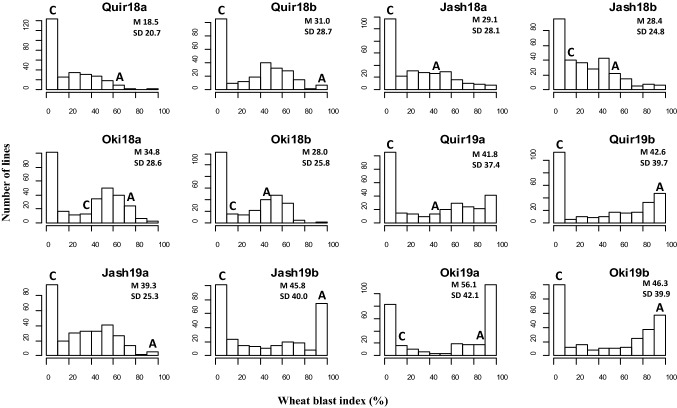
Histograms of wheat blast index in the Caninde#1/Alondra population in individual environments. Phenotypic ranges of the two parents are indicated, where *C* stands for Caninde#1 and *A* for Alondra. ‘Quir’ stands for Quirusillas, ‘Jash’ for Jashore and ‘Oki’ for Okinawa, ‘18′ and ‘19′ for the 2017–2018 or 2018 cycle and 2018–2019 or 2019 cycle, respectively, and ‘a’ and ‘b’ for the first and second sowing, respectively. Grand mean (M) and standard deviation (SD) values are presented for all experiments

WB index varied greatly across the 12 environments, with Quir18a being the lowest with a grand mean of WB index of 18.5% and Oki19a the highest with 56.1%. The disease variation corresponded generally well with the climatic data, where warmer and higher precipitation in February (Quirusillas and Jashore) or July (Okinawa) favored WB infection (Fig. S1). The resistant parent Caninde#1 showed consistently lower WB infection than the susceptible parent Alondra, with their respective WB index ranged from 0 to 32% and 45 to 100% across environments. Transgressive segregation was often observed, in both the resistant and susceptible directions (Fig. 1). ANOVA indicated significant effects of ‘Genotype’ as well as those of ‘Genotype × Year’ in all three locations, and moderately high heritability estimates were obtained that ranged from 0.71 for Quirusillas to 0.87 for Okinawa (Table 1). Phenotypic correlations of WB were all significant among experiments, with *r*-values ranging from 0.41 to 0.87. In general, higher correlation coefficients were found among experiments in Bolivia, whereas lower correlation coefficients were observed among experiments in Bangladesh (Table 2).

**Table 1:** Analysis of variance for wheat blast index in different locations and its heritability estimates

Location	Source	DF	Mean square	*F* value	*P* value	Heritability
Quirusillas	Genotype	297	2641.00	10.68	< 0.0001	0.71
	Year	1	74,437.42	301.01	< 0.0001	
	Sowing (year)	1	8059.75	32.59	< 0.0001	
	Genotype × year	287	721.37	2.92	< 0.0001	
	Genotype × sowing	291	310.66	1.26	0.0336	
	Error	238	247.30			
Jashore	Genotype	297	2256.78	5.67	< 0.0001	0.74
	Year	1	31,555.83	79.26	< 0.0001	
	Sowing (year)	1	14,699.25	36.92	< 0.0001	
	Genotype × year	297	571.83	1.44	0.0009	
	Genotype × sowing	297	421.93	1.06	0.3086	
	Error	297	398.13			
Okinawa	Genotype	297	3912.81	24.09	< 0.0001	0.87
	Year	1	117,455.51	723.16	< 0.0001	
	Sowing (year)	1	342.04	2.11	0.1478	
	Genotype × year	294	440.79	2.71	< 0.0001	
	Genotype × sowing	297	247.29	1.52	0.0002	
	Error	282	162.42

**Table 2:**
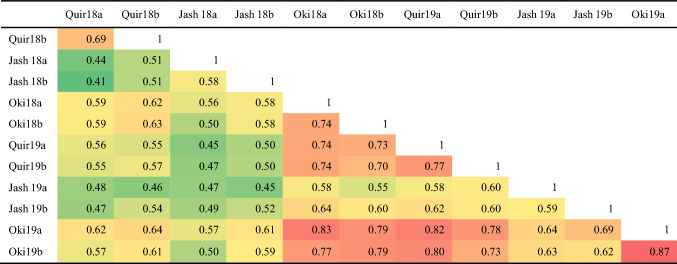
Pearson's correlation coefficients of wheat blast index among the 12 environments (color table online)

All correlations were significant at *P* < 0.0001. ‘Quir’ stands for Quirusillas, ‘Jash’ for Jashore and ‘Oki’ for Okinawa, ‘18′ and ‘19′ for the 2017–2018 or 2018 cycle and 2018–2019 or 2019 cycle, respectively, and ‘a’ and ‘b’ for the first and second sowing, respectively. Cell shades change from green to red with the increase of correlation coefficients

In Okinawa, late-sown experiments exhibited consistently lower WB than the earlier-sown ones, whereas the trends were less clear in Quirusillas and Jashore (Fig. 1). Correlation of WB with DH and PH was either nonsignificant or significant at low levels (Table 3). Of the significant correlations between WB and DH, those in Jashore experiments were always positive, whereas those in Quirusillas and Okinawa experiments were all negative, except for Quir18a. In the case of PH, however, all significant correlations were negative (Table 3), implying that tall plants tended to have a low level of WB.

**Table 3:** Phenotypic correlation of wheat blast index with days to heading (DH) and plant height (PH) in individual environments

	Quir18a	Quir18b	Jash18a	Jash18b	Oki18a	Oki18b	Quir19a	Quir19b	Jash19a	Jash19b	Oki19a	Oki19b
DH	0.15*	0.03	0.21**	0.03	0.07	− 0.05	− 0.25**	− 0.14	0.2**	0.17**	− 0.06	− 0.21**
PH	0.04	− 0.08	− 0.32**	− 0.26**	− 0.04	− 0.07	− 0.14*	− 0.02	− 0.08	− 0.19**	− 0.26**	− 0.36**

‘Quir’ stands for Quirusillas, ‘Jash’ for Jashore and ‘Oki’ for Okinawa, ‘18′ and ‘19′ for the 2017–2018 or 2018 cycle and 2018–2019 or 2019 cycle, respectively, and ‘a’ and ‘b’ for the first and second sowing, respectively

*p < 0.01; ** *p* < 0.001

### Genotyping and linkage analysis

Initially, 78,255 SNP markers were scored for this population, and finally, 2131 non-redundant markers of high quality were used for subsequent analysis. Thirty-six LGs were generated, representing all 21 wheat chromosomes, of which 2B had the highest number of markers (301) and 4D had the lowest number of markers (16), whereas only four markers remained unlinked. These LGs covered a total genetic distance of 4851 cM, with an average distance between markers of 2.3 cM.

### QTL mapping

**Fig. 2:**
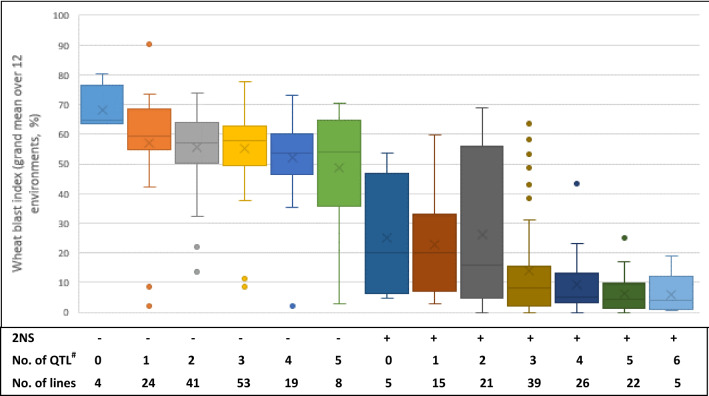
Phenotypic effects of 2NS and its combinations with different numbers of minor QTL identified in this study. The allelic status of 2NS was determined by the two flanking markers 3958902 and 1209870, and other markers in the QTL region as well as phenotypic data were also considered when missing or recombination happened between the two markers. Cross and horizontal lines in each box denote the mean and median values, respectively. # only non-2NS QTL were counted

Seven QTL have been identified on chromosomes 1AS, 2AS, 2BL, 3AL, 4BS, 4DL and 7BS, of which only the one on 2NS/2AS was consistently significant across environments, explaining phenotypic variation from 22.4 to 50.1%, whereas other QTL were of minor effects and were significant in only a subset of experiments (Table 4). The QTL on 7BS was significant in nine out of 12 experiments, being the second most stable QTL and explaining phenotypic variation from 3.7 to 7.4%. The remaining minor QTL were significant in two (1AS) to six (4BS) experiments. Caninde#1 contributed resistant alleles of the QTL on 2NS/2AS, 1AS and 4DL, whereas the susceptible parent Alondra contributed those of the remaining four QTL (Table 4). It is noteworthy that the QTL on 4BS was linked to a QTL for PH in the *Rht-B1* region (Fig. S2), whereas none of the remaining QTL showed any association with PH or DH (data not shown). Boxplot chart showing phenotypic effects of stacking different QTL exhibited clear-cut results between groups with and without 2NS, demonstrating the dominant role of 2NS in conferring WB resistance in this population (Fig. 2). Within 2NS or non-2NS group, however, minor QTL showed additive effects, i.e., the more QTL stacked, the lower the average or median WB index (Fig. 2).

**Table 4:** Phenotypic effects (%) of QTL for wheat blast index across 12 environments

Chr	Position	Left marker	Right marker	Quir18a^a^	Quir18b	Jash18a	Jash18b	Oki18a	Oki18b	Quir19a	Quir19b	Jash19a	Jash19b	Oki19a	Oki19b	R source^b^
1AS	0.7–1.5	1043336	5370345					4.1			4.0					C
2AS	3.6–4.8	3958902	1209870	**22.4**	**30.1**	**22.6**	**22.8**	**38.8**	**39.1**	**40.3**	**35.3**	**22.5**	**30.3**	**30.1**	**50.1**	C
2BL	305.5–308.3	3064531	2269494					3.7		3.7		3.5		4.4		A
3AL	33.1–41.8	1088469	1242936						4.5	4.6	3.5			3.7	4.2	A
4BS^c^	36.6–51.2	1151382	6027240				**4.9**		3.5	**7.0**		3.0		4.0	4.8	A
4DL	0.0–22.6	1125184	1230255	4.1					3.5		4.1					C
7BS	35.5–46.8	1228818	1001752	4.1			4.2		**7.4**	**6.4**	**6.9**	**4.5**	**7.3**	**4.9**	3.7	A
Accumulated percentage of variation explained				30.6	30.1	22.6	31.9	46.6	58.0	62.0	53.8	33.5	37.6	47.1	62.8	

^a^The percentages of phenotypic variation explained are shown in the table. QTL with LOD values higher than 2 are listed, and those with LOD higher than 3 are bolded***. ***‘Quir’ stands for Quirusillas, ‘Jash’ for Jashore and ‘Oki’ for Okinawa, ‘18′ and ‘19′ for the 2017–2018 or 2018 cycle and 2018–2019 or 2019 cycle, respectively, and ‘a’ and ‘b’ for the first and second sowing, respectively

^b^***C*** for Caninde#1 and ***A*** for Alondra

^c^This QTL is linked to a QTL for plant height in the *Rht-B1* region

### The 2NS QTL and its associated markers

**Fig. 3:**
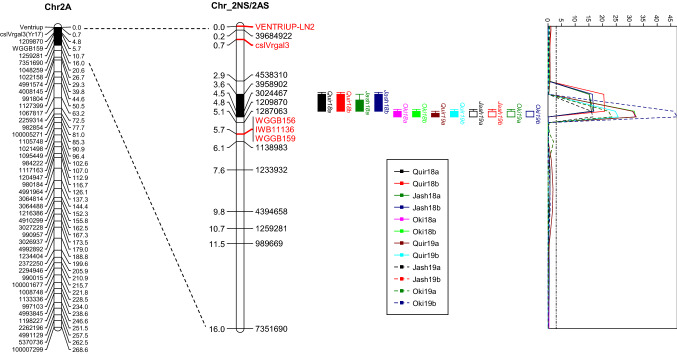
QTL profile for wheat blast resistance on chromosome 2NS/2AS across environments. Genetic distances are shown in centimorgans to the inner sides of the linkage groups (LG). Only framework markers are presented on the 2A LG, whereas all mapped markers are shown on the 2NS/2AS LG, where non-SNP markers are highlighted in red. A threshold of 3.0 is indicated by a dashed vertical line in the LOD graph. The profiles of the remaining QTL are presented in Fig. S2 (color figure online)

The 2NS/2AS translocation segment corresponds to a 16.0-cM region on the distal part of the 2A LG, and the WB-resistant QTL was mapped between DArTseq markers 3958902 and 1209870, corresponding to a 1.2-cM region (Fig. 3). The five STS markers were found in the flanking regions of this QTL, with *Ventriup-LN2* and *cslVrgal3* in its distal region with genetic distances of 3.6 and 2.9 cM, respectively, and the three co-segregating markers *WGGB159*, *WGGB179* and *IWB11136* in its proximal side with a genetic distance of 0.9 cM (Fig. 3).

## Discussion

WB resistance of a wheat line can vary greatly under different environmental conditions; thus, an appropriate disease pressure is needed to clearly differentiate the genotypes under evaluation. Mostly, WB pressure under natural infection is insufficient to correctly assess lines for their resistance levels. Early breeding work for WB resistance in South America relied mostly on natural infection, which happened sporadically across years and often unevenly in the experimental fields, enabling some genotypes to escape the infection and appear resistant or tolerant (Kohli et al. 2011; Duveiller et al. 2016). This, along with the gain of virulence of newly emerged MoT isolates, could be the most important reasons explaining that early-suggested WB resistant lines turned out to be susceptible in later evaluations. In the current study, the first field evaluation took place in the 2017 cycle in Okinawa (denoted as Oki17), under natural infection. However, the WB infection was very weak, with about half of the population showing no symptom and a grand mean of WB index of merely 1.9%, and there was no significant correlation between Oki17 and those presented in Table 2. Likewise, the 2NS/2AS QTL for Oki17 was of minor effect with phenotypic effect of only 7.0% (data not shown), much smaller than from other environments. Therefore, Oki17 was not used in this study. Quirusillas is another hot spot of WB in Bolivia, where researchers and breeders have set up WB screening nurseries. Some breeding materials were sown there in the 2016–2017 cycle without artificial inoculation nor misting system, and the results turned out to be unsatisfactory (data not shown). This shows that artificial inoculation and misting are indispensable for obtaining robust phenotypic data of WB, even in hot spots like Quirusillas and Okinawa. Therefore, artificial inoculation and misting have been adopted in the two screening sites in Bolivia since 2018, as well as in Jashore, Bangladesh, upon the establishment of WB screening nursery in the 2017–2018 cropping cycle. It is noticeable that even with artificial inoculation and misting system, WB development was not very high in several environments, like Quir18a and Jash18b (Fig. 1). Considering also the significant genotype-by-environment interaction, field evaluation for WB must be performed in multiple environments (preferably in different locations across years) in order to obtain robust phenotypic results.

It is well accepted that early-sown materials usually get heavier WB infection than the late-sown ones in South America; thus, it is not suggested to plant wheat too early in order to avoid the WB infection (Goulart et al. 1990; Cruz and Valent 2017). This rule, however, was based on prevailing weather conditions, whereas in a specific cropping cycle it may not be true. For example, during the 2017–2018 cropping season in Quirusillas, a reverse trend was observed. Nevertheless, data from Okinawa agreed well with the general trend (Fig. 1). In South Asia, temperature and rainfall during the late part of the wheat season are expected to increase and become more conducive for WB; thus, late-sown or late-headed lines are expected to have more severe WB infection, as shown by our results (Table 3, Fig. 1). Therefore, a recommendation to South Asian farmers in terms of WB would be to avoid planting the crop late, which agrees well with the avoidance of spot blotch and terminal heat (Joshi et al. 2007).

Plant height has shown a close association with Fusarium head blight (FHB), a major spike disease of wheat, with one of the underlying mechanisms being that spikes of tall plants are well ventilated and thus become drier and have lower FHB severity, compared to those of short plants (Yan et al. 2011). Considering the likewise requirement of WB for high humidity, a similar association between WB and PH is expected, which was demonstrated for the first time in the current study (Table 3). Notably, the WB QTL on 4BS partly overlapped with a PH QTL at *Rht-B1* (Fig. S2), and this genetic linkage might have contributed to the phenotypic correlation. Apart from this QTL, none of the remaining QTL was associated with PH, being markedly different from those for FHB (He et al. 2016; Xu et al. 2020). This might also imply that PH has less impact on WB than on FHB, but further study is needed to corroborate or negate it.

As expected, the 2NS/2AS translocation demonstrated consistent and large phenotypic effects on reducing WB infection, but its effects were not high enough to be regarded as a simple Mendelian factor. This implies that 2NS alone cannot provide complete protection against WB, which is in agreement with Cruz et al. (2016b). In fact, the most susceptible 2NS carrier showed a grand mean of WB index close to 70%, and many had a grand mean higher than 50% (Fig. 2), showing that 2NS-mediated resistance is highly dependent on the genetic background, where other factor(s) might be required to fulfill its function. The breakdown of 2NS resistance to WB has been reported in Paraguay (Singh et al. 2016) and Brazil (Ceresini et al. 2018). In a greenhouse experiment conducted in Bolivia, the well-known 2NS carrier Milan exhibited an average WB severity of 35% (Marza et al. 2019). Therefore, 2NS resistance is most likely incomplete, at least under high WB pressure, and new MoT isolates appear to have higher virulence to the 2NS carriers (Cruz et al. 2016b). To achieve better and durable resistance, additional QTL are necessary. As shown in our results, the more QTL accumulated, the less WB is observed. Unfortunately, all non-2NS QTL identified in this study were of minor effects and would be difficult to use in a MAS strategy. It is imperative to identify novel QTL with major effects for WB resistance, so that they can be utilized in breeding to reduce the high selection pressure 2NS is exerting on the MoT population. In this regard, non-2NS genotypes identified by Cruppe et al. (2020) as resistant to WB could be analyzed in genetic studies for the discovery of new QTL. Nevertheless, without such QTL, it is still possible to obtain non-2NS lines with an acceptable level of WB resistance by stacking multiple minor QTL. Such examples can be observed from Fig. 2, where many non-2NS lines showed good WB resistance, although we should keep in mind that a few of them might be actually 2NS carriers that were mistakenly classified as non-2NS based on flanking markers, because no functional marker is currently available for this QTL. The strong phenotypic effects of multiple minor genes were well demonstrated in wheat rusts (Singh et al. 2016), but we must say that to do the same in WB is far more challenging, considering the difficulty in WB phenotyping and the small phenotypic effects of the so-far known minor QTL.

The molecular marker *Ventriup-LN2* has been widely used for diagnosing the presence of the 2NS segment in hexaploid wheat with good accuracy (Helguera et al. 2003; Cruz et al. 2016b). The drawback of this marker is its dominant nature, which might lead to false-negative results when DNA quality is poor. The marker amplifies PCR product in 2NS carriers; thus, it cannot differentiate heterozygous (2NS/2AS) from homozygous (2NS/2NS) genotypes. Additionally, this marker is distal to the WB resistance QTL, with a distance of 3.6 cM in the current mapping population, making it less diagnostic. The marker *cslVrgal3* is similar to *Ventriup-LN2* in all aspects. Different to these two markers, *WGGB156* and *WGGB159* developed by Wang et al. (2018b) are codominant, closer to the QTL, and thus could be more effective for tracking the QTL in crosses.

The WB resistance QTL from 2NS has a projected physical region of 2.3 Mb on the 2AS chromosome of Chinese Spring (IWGSC RefSeq v1.0). This region harbors 79 annotated high-confidence genes, of which 14 are of NBS-LRR gene family, three are of disease resistance protein, as well as genes belong to receptor-like protein kinase, ABC transporter G family member, dirigent protein, F-box domain protein and defensin that have been associated with disease resistance in wheat. Nevertheless, the underlying gene for this QTL might not be one of them, since Chinese Spring is a 2AS carrier, whereas the resistance allele of this QTL comes from 2NS. However, the clusters of resistance genes in the reference genome may indicate that 2NS/2AS QTL region is enriched for resistance-encoding genes, which could be a hint for future investigation of the locus.

## Electronic supplementary material

Below is the link to the electronic supplementary material.

Supplementary file 1 (DOCX 254 kb)
