# Supplementary material for: QTL mapping for field resistance to wheat blast in the Caninde#1/Alondra population
Source: Theor Appl Genet. 2020 Jun 2;133(9):2673–83. doi: 10.1007/s00122-020-03624-x (PMC7419448; doi:10.1007/s00122-020-03624-x)
Supplement: Supplementary file 1 — (DOCX 254 kb) [file 122_2020_3624_MOESM1_ESM.docx]

**Rain fall (mm)**

**Temperature**


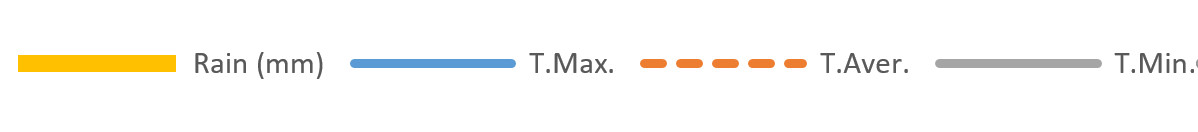


**Fig. S1** Monthly weather data for Okinawa (the 2018 and 2019 cropping seasons), Quirusillas (the 2017-18 and 2018-19 seasons) and Jashore (the 2017-18 and 2018-19 seasons).

**Fig. S2** QTL profiles for wheat blast resistance in the Caninde#1/Alondra population across environments. Genetic distances are shown in centimorgans to the left side of the linkage groups (LG). Only framework markers are presented except for the QTL region where all markers are shown. A threshold of 2.0 is indicated by a dashed vertical line in the LOD graph
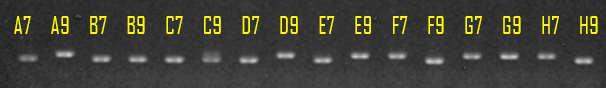

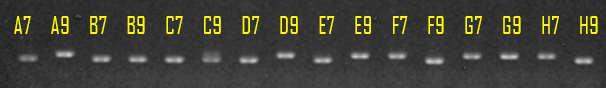

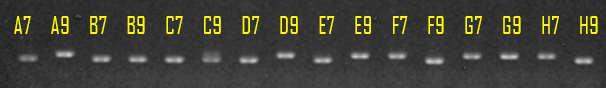

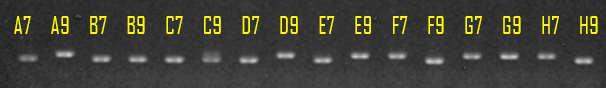

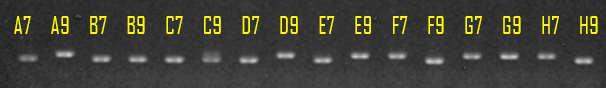

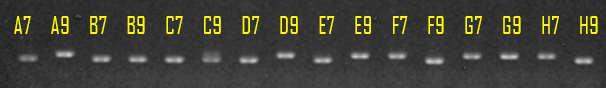
. The 2NS/2AS QTL profile is presented in Fig. 3.
